# How to reduce ‘double-firing’-induced scope damage by investigating the relationship between laser fiber core degradation and fiber jacket burn?

**DOI:** 10.1371/journal.pone.0233135

**Published:** 2020-05-22

**Authors:** Seung Hoon Ryang, Tam Hoai Ly, Hyun Sik Yoon, Dae Hyoung Park, Sung Yong Cho

**Affiliations:** 1 Department of Urology, Seoul National University Hospital, Seoul, South Korea; 2 Department of Urology, Cho Ray Hospital, Ho Chi Minh City, Viet Nam; University of Vigo, SPAIN

## Abstract

**Purpose:**

'Double-firing effect' in which laser firing occurs in the fiber tip and its proximal part is caused by different breakdown rates between fiber jackets and cores. This study investigated a new safe distance concept to prevent scope damage by analyzing the breakdown of the laser fiber jacket and cores.

**Methods:**

Laser fibers were fixed in a benchtop simulation model. The fiber tip was in contact with uniform phantom stones and submerged in saline. Four different energy settings (1.0 or 2.0J x 10Hz or 30Hz) and two different fiber sizes (200 μm and 365 μm) were tested. After three minutes of use at each energy setting, the length of fiber shortening and jacket burn were measured. The fibers were stripped to measure the length of core degradation.

**Results:**

Mean degradation lengths were 4.2 to 7.8 mm. There was no statistical difference in the mean lengths of fiber core degradation and jacket burn. However, core degradation was longer than the jacket burn in half of the samples. The mean difference in lengths between core degradation and jacket burn was 0.49 ± 0.90 mm. Lengths of core degradation and the jacket burn were longer at the setting of high-power energy and 200 μm fiber - 2J with 30 Hz.

**Conclusion:**

To reduce ‘double-firing’-induced damage, the authors recommend that laser fiber should be cut 1.0 mm longer than visible jacket burn at high-power settings after 3-min continuous fragmentation. After cutting the fiber, the laser should be checked whether ‘double-firing’ is no more seen.

## Introduction

The indications for the use of flexible ureteroscopes (fURS) are broadening in retrograde intrarenal procedures because of technical improvements in the instrument. Due to its flexibility, fURS can be used for diagnostic procedures and the treatment of complex stones or malignancies. Although decreasing the diameter of the scope’s working segment makes fURS more fragile than before, the tip size of fURS should be restricted in order to reach the upper ureter and renal pelvis without ureter trauma.

**Fig 1:**
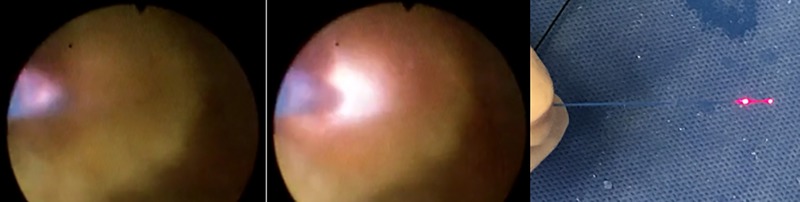
Double firing effect. The laser is firing at both the tip and proximal parts of the fiber.

The most common cause of fURS failure is working channel breakdown related to the laser. [1, 2] If the laser is fired too close to the instrument, the fURS can be damaged by cavitation bubbles and high temperatures. [3] No matter how carefully the operator tries to fire the laser at a distance from the scope, laser fiber tip degradation can occur due to the continuous firing of the laser. Previous researchers have referred to this phenomenon as ‘burn back’. [4] Therefore, the laser can be fired unintentionally in a close range to the instrument from the laser fiber that is degraded during the procedure. Talso and coworkers have already reported on a ‘safety distance concept’ for laser fiber tips and fURS. [5] However, a laser is often fired not only on the tip, but also on its proximal part. The authors of the present study call it ‘double-firing’ (Fig 1). It may mean that the length of the laser fiber jacket burn can be different from that of the laser fiber core degradation.

Therefore, the present study aimed to establish a new safe distance concept to prevent scope damage by investigating the relationship between fiber jacket burn and fiber core degradation. The authors investigated whether the length of the fiber burnt jacket can be the standard point for cutting and it would be helpful to prevent from ‘double-firing effect’.

## Materials and methods

The goal of this experiment was to measure the length of fiber jacket burn and fiber core degradation in different energy settings of 100W Ho:YAG laser without MOSES technology (Versapulse power suite, Lumenis, Yokneam, Israel). Pulse widths were normally around middle (750 μsec). The tips of the laser fibers (Boston Flexiva ^TM^, Boston Scientific, Marlborough, MA, USA) were cut and collected after three minutes of stone fragmentation. A new single-use 200 μm or 365 μm laser fiber (Boston Scientific) was placed in the self-made benchtop model. The authors tried 1, 2, 3, 4, and 5 min of laser firing in the first place. The baseline information was that the laser fiber length outside of the flexible ureteroscope was approximately 3–4 mm when the authors reviewed the flexible ureteroscopic and the percutaneous nephroscopic views. After 1 and 2 minutes, the laser fiber degradation and jacket burn were short. Finally, the authors decided to perform this experiment after 3 min of laser firing.

The fiber tip was in contact with uniform phantom stones. The phantom stones were made with a powder-to-water mixing ratio of 15:3, producing a 3 mm-sized stone with properties similar to calcium oxalate monohydrate. To maintain contact between the phantom stones and the laser fiber tip, the laser fiber was held by hand during stone fragmentation. The researchers found that holding the laser fiber in hand during the laser firing and following the movement of the artificial stone was more similar to actual endoscopic surgery than affixing the laser fiber to the benchtop model.

### Benchtop model

**Fig 2:**
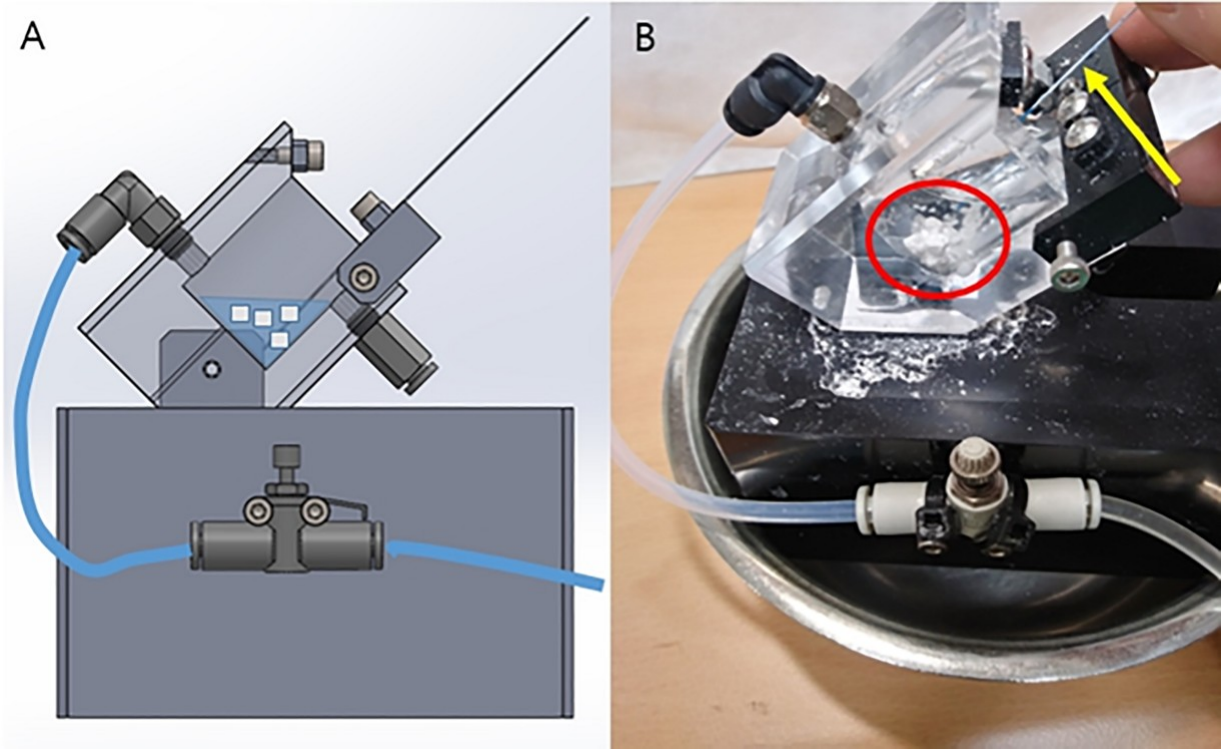
(A) Diagram of experimental benchtop setup. (B) Actual experiment scene. Artificial stones with properties similar to calcium oxalate monohydrate are placed in clear plastic tubing. (Circle) Laser fiber is hand-held and the tip of fiber is nearly contact to artificial stones. To measure the shortened length, the laser fiber is marked every 3cm with a white marker. (Arrow).

To evaluate the shortening of laser fiber during lithotripsy, the authors designed the benchtop model shown in Fig 2. The angle was around 25 degrees which mimicked the upper pole calyx in the kidney when the patients lie in the Trendelenburg position. To reproduce conditions similar to intra-renal surgery, the fiber tip and phantom stones were placed in clear plastic tubing (inner diameter = 3 cm) and submerged in 0.9% saline, which is drained using a thin plastic tube inside the bench top model. Four pieces of phantom stones were initially used for stone fragmentation in each setting. The laser fiber can be fixed at the entrance of the plastic tubing space under the screw (yellow arrow). The material was all waterproof plastic.

### Laser fiber collection

The authors marked 3 cm from the fiber tip prior to lithotripsy. Four different energy settings (1.0Jx10Hz, 1.0Jx30Hz, 2.0Jx10Hz, and 2.0Jx30Hz) and two different fiber sizes (200 μm and 365 μm) were tested. After three minutes of use at each energy setting, the laser fiber tip was cleaved using CS‐124 ceramic scissors (Kyocera, Kyoto, Japan). To eliminate the possibility of laser fiber damage due to cutting with the scissors and to measure the shortened length of the laser fiber, the authors cut the fiber at the previously marked site.

### Inspection of the shortened laser fiber

**Fig 3:**
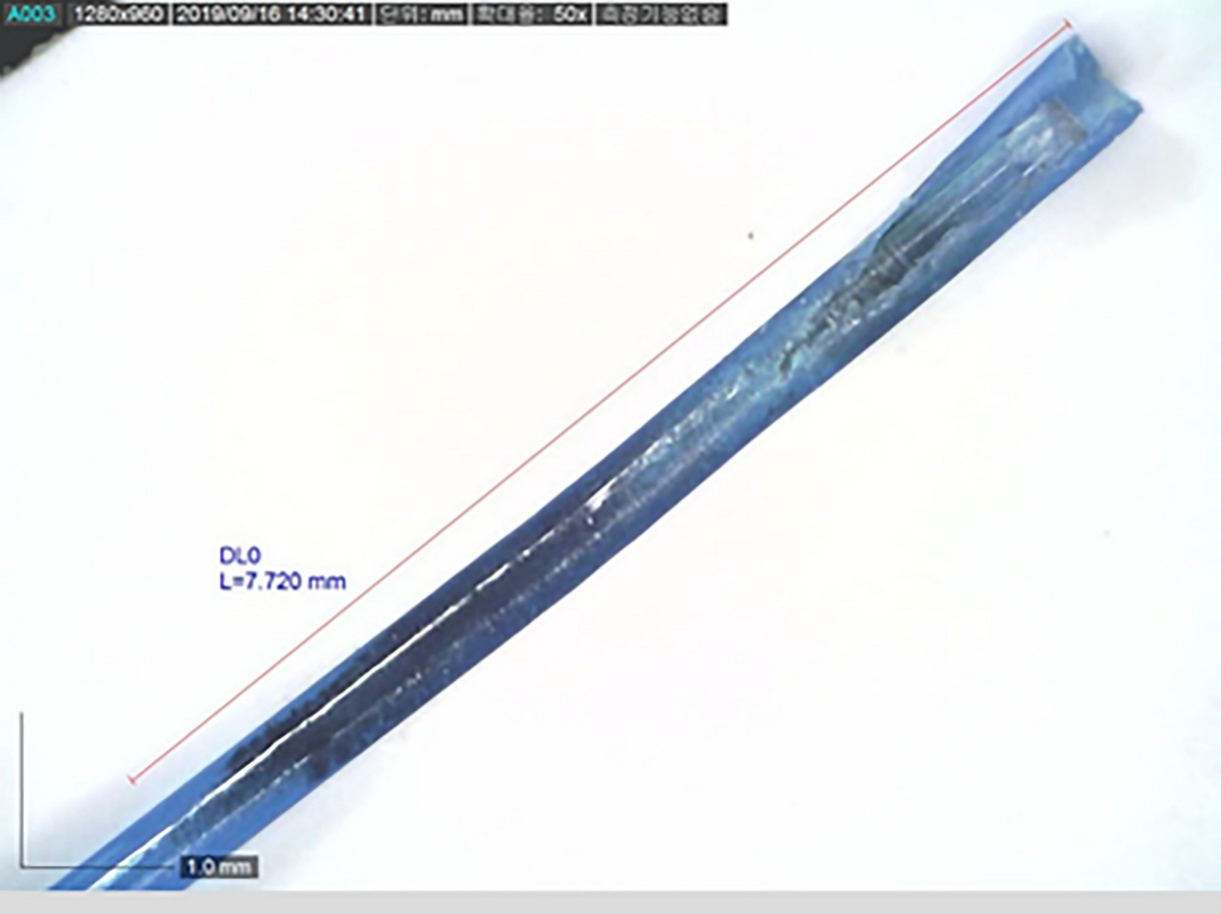
Measurement of burnt length of jacket fiber.

**Fig 4:**
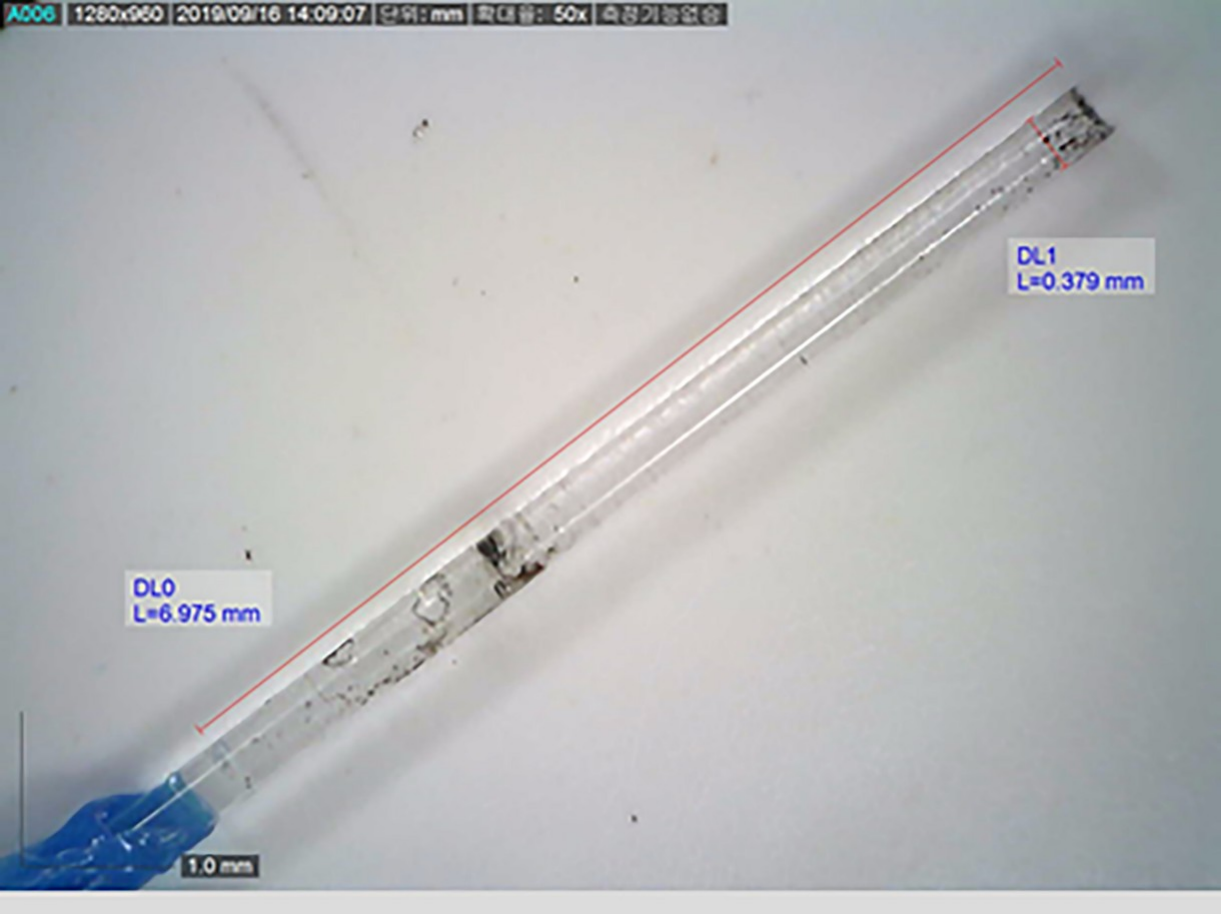
Fiber was stripped to measure the length of fiber core degradation.

All collected fibers were inspected in a prescribed order. First, the length between the laser fiber tip and a 3-cm marking was measured to check the shortened length of the laser fiber. The burnt length of the fiber jacket was also measured. The length was measured under 230 X magnification using a digital microscope (AD7031MZT, Dino-lite, Taipei, Taiwan) (Fig 3). Jacket fiber was then stripped to 200 μm or 365 μm with a Fiber Stripper (Boston Scientific). The stripped fiber was microscopically inspected to measure the fiber core degradation length (Fig 4).

### Statistical analysis

An independent Mann-Whitney U test was performed to determine the relationship between degradation of the fiber jacket and the fiber core. Multivariable linear regression analysis was performed to determine the factors affecting the length of the fiber jacket burn and core degradation. Statistical significance was defined at *p* < 0.05. All statistical analyses were conducted using IBM SPSS version 24.0 Software (IBM, Armonk, NY, USA).

## Results

After three minutes of laser firing, almost all stones were totally fragmented to less than 1 mm in size. As shown in Table 1, the shortened lengths of the laser fiber and fiber jacket burn were affected by both the fiber caliber and the laser power setting of pulse energy and frequency. In particular, pulse energy (*p* <0.001 in core degradation, *p* = 0.019 in jacket burn) had a greater effect on fiber shortening than other factors, such as laser frequency (*p* = 0.004 in core degradation, *p* = 0.001 in jacket burn) and fiber caliber (*p* = 0.019 in core degradation, *p* = 0.002 in jacket burn).

**Table 1:** Multivariable linear regression model: Predictive factors of degradation length of fiber core and fiber jacket burn.

Laser core degradation (R^2^ = 0.381 Adjusted R^2^ = 0.357 F^2^ = 38.33 P value<0.001)					
	B	SE	β	T	P value
Coefficient	0.345	0.888		0.389	0.698
Fiber caliber (μm)	0.005	0.002	0.217	2.406	0.019*
Energy (J)	1.986	0.350	0.512	5.671	<0.001*
Frequency (Hz)	0.052	0.018	0.269	2.986	0.004*
**Fiber jacket burn** (R^2^ = 0.481 Adjusted R^2^ = 0.461 F^2^ = 45.59 P value<0.001)					
	B	SE	β	T	P value
Coefficient	-0.272	0.790		-0.345	0.731
Fiber caliber (μm)	0.006	0.002	0.269	3.257	0.002*
Energy (J)	2.176	0.312	0.577	6.985	<0.001*
Frequency (Hz)	0.052	0.016	0.275	3.326	0.001*

* P<0.05

With the use of 200 μm laser fiber, the length of the core degradation and jacket burn showed no significant difference at low power settings (1J with 10Hz, 1J with 30Hz, and 2J with 10Hz). However, the length of the fiber core degradation was significantly longer than that of the jacket burn in the high power setting of 2J with 30Hz (7.51±1.72 mm vs 7.21±1.60 mm, *p* = 0.028). When comparing the shortened length according to power setting in post-hoc analysis with the Bonferroni method, the degradation lengths of the fiber core and jacket burn did not show any significant differences across the low power settings of 1J with 10Hz, 1J with 30Hz, and 2J with 10Hz (Table 2).

**Table 2:** Mean length of degradation of fiber core and jacket burn after three minutes of use.

	Core degradation(mm)	Jacket burn (mm)	P value
**200 μm**			
(A) 1.0 J x 10 Hz (n = 10)	4.21±0.86	4.35±0.68	0.652
(B) 1.0 J x 30 Hz (n = 10)	5.20±1.68	4.69±1.03	0.234
(C) 2.0 J x 10 Hz (n = 10)	4.63±1.57	4.77±1.64	0.137
(D) 2.0 J x 30 Hz (n = 10)	7.51±1.72†	7.21±1.60†	0.028*
**365 μm**			
(A) 1.0 J x 10 Hz (n = 10)	4.75±1.33	4.25±0.84	0.249
(B) 1.0 J x 30 Hz (n = 10)	5.11±1.61	5.40±1.25	0.270
(C) 2.0 J x 10 Hz (n = 10)	**7.55±0.97**‡	**7.60±1.36**‡	0.834
(D) 2.0 J x 30 Hz (n = 10)	**7.51±1.67**‡	**7.82±1.61**‡	0.270

* P<0.05

† Degradation of core (P<0.001*) & jacket (P <0.001*) in 200 μm laser fiber: A = B = C<**D**

‡ Degradation of core (P<0.001*) & jacket (P <0.001*) in 365 μm laser fiber: A = B<**C** = **D**

In the analysis of the 365 μm laser fiber, there was no significant difference in the length of core degradation or jacket burn at any power setting. A comparison of the breakdown lengths between the different power settings showed that the length of the fiber core degradation and jacket burn at the 2J setting was significantly longer than that at the 1J settings (*p* < 0.001) as shown in the Table 2.

The average value of the total data showed no statistical difference between the lengths of fiber core degradation and jacket burn (5.81 ± 1.89 mm vs. 5.76 ± 1.95 mm, *p* = 0.627). However, the length of fiber core degradation was longer than that of the jacket burn in 50% (40 of 80) of the samples. The mean difference in lengths between core degradation and jacket burn was 0.49 ± 0.90 mm in the samples.

## Discussion

To the best of our knowledge, the present study showed the correlation between shortening the laser fiber jacket, burn-back and fiber core degradation during lithotripsy for the first time. This study showed the real situation of laser-induced fiber damage during renal stone surgery by mimicking the most commonly encountered situations with scope damage. Therefore, the results of the present study can be applied to the renal clinical practice during renal stone surgery.

fURS is a very delicate instrument due to the narrow diameter of its working elements. This makes fURS notorious for its limited durability and high cost of repair. Afane et al. [6] reported that a fURS smaller than 9 Fr required major repairs after six to 15 procedures or three to 13 hours of use. The cost of fURS damage is very high. If 100 cases of fURS lithotripsy are performed without warranty, the cost of repairing the scope is 46–59% of the total cost [2]. The average cost per repair of a fURS is 4,597 US dollars [7], which discourage many surgeons. Although there are some differences in figures, several studies have shown that laser-related injury is the single most common cause of fURS damage. Sung et al. [7] reported that 42% of the working channel damage and 45% of the deflection component failures were caused by errant firing of the laser. In a single tertiary center experience report, 11 of 14 fURS breakdown cases that needed repair were laser-related. [8] There is also a report that the use of laser was more likely to cause damage to the fURS than non-energy modalities, such as endoluminal graspers. [9]

Several studies have established strategies to prevent energy leaks and the resulting fURS damage. Daniel [10] and coworkers conducted an *in vitro* study on the correlation between energy setting and the degradation length of laser fiber. Their study demonstrated that low energy levels with high-frequency setting could reduce stone retropulsion and laser fiber tip degradation while maintaining stone fragmentation efficiency. Kronenberg and Traxer [11] showed that stripping fibers, the use of harder stone material, and high pulse energy settings were associated with increased fiber tip degradation. They also reported that cutting the laser fiber with metallic or ceramic scissors was simpler and safer than the stripping method. Talso [5] and coworkers suggested a “safety distance concept”. To prevent scope damage, they recommended positioning the laser fiber tip at least one quarter the distance away from the monitor when firing the laser.

In our institution, hundreds of endoscopic laser lithotripsies are performed every year and a significant portion of them are complicated cases requiring prolonged operation time. For this reason, laser fiber tip degradation is a major concern for us. Although many previous studies have established the concept of laser fiber tip degradation, the authors have not considered it to be sufficient for the safe use of fURS. When using the laser for a long time during laser lithotripsy, firings are often seen in the proximal part of the fiber tip, not only in the laser fiber tip (Fig 1). This could represent the difference of burn-back between the laser fiber jacket and the fiber core. The authors consider this effect as a potential risk factor to damage the scope by causing unintended close laser firing during the procedure. Therefore, this study was conducted to determine the difference in the degradation length of the jacket and the core of the laser fiber tip.

Consistent with previous studies, the data from this study showed that high energy settings caused more fiber core degradation and jacket burn than low energy settings. Overall, the fiber core degradation and the shortened length of the fiber jacket showed a good correlation. However, it was confirmed that fiber core degradation could occur faster than jacket burn in the 200 μm laser fiber at a high energy setting of 2J with 30Hz. This suggests that when using small caliber laser fiber at a high-power setting, it is necessary to cut 1 mm more than the visible burned fiber portion to prevent scope damage due to double-firing. For 365 micrometer fiber, there was no significant difference between fiber core degradation and jacket burn. Therefore, it would be sufficient to cut the visible burned fiber portion when using a large caliber fiber.

Although no statistical difference was found between the lengths of laser fiber core degradation and jacket burn (5.81 ± 1.89 mm vs. 5.76 ± 1.95 mm, *p* = 0.627), the length of laser fiber core degradation was longer than that of jacket burn in 50% (40 of 80) of the samples. The mean difference in lengths between the core degradation and jacket burn was 0.49 ± 0.90 mm. Thus, the authors of this study suggest that it is necessary to cut 1–1.5mm more than the visible burned portion of the laser fiber jacket after three minutes of the pop-dusting technique regardless of the laser fiber caliber or power setting to prevent scope damage caused by the double-firing effect.

Another interesting result of this study was that the core degradation length of the 365 μm fiber at a 2J-10Hz energy setting was longer than that of the 200 μm fiber (365 μm vs. 200 μm: 7.55 ± 0.97mm vs. 4.63 ± 1.57 mm, *p* < 0.001). This is thought to be because the efficiency of the laser resonator's energy concentration to the fiber is reduced when a small caliber fiber is used. Based on this finding, we can assume that the stone fragmentation efficiency of the 200 μm fiber is lower than that of the 365 μm fiber at the 2J-10Hz energy setting. However, further study is needed to validate this.

This study had several limitations. First of all, because data in this study were obtained from benchtop model experiments, it was impossible to reproduce all physiological factors that occurred when performing lithotripsy in living patients and the results can be affected by surgeons’ experience. [12] The findings of this study should be proven through a well-designed clinical trial. Second, this study was limited to artificial stone mimicking a single stone type. However, the length of the laser breakdown depends on the type of stone. Spore [13] and coworkers reported that uric acid and cystine stones can cause less laser fiber tip degradation. However, the phantom stones that the authors used only mimicked the physical properties of calcium oxalate monohydrate stone. [14] Third, only two sizes of laser fiber produced by a single manufacturer were included in this study. Adam [15] and coworkers showed that fiber shortening and energy transmission vary among fibers from different manufacturers. Therefore, additional studies on various stone types and laser fiber types should be conducted. Lastly, it is necessary to introduce some reference to the work where this effect was studied in depth and where the double-firing effect is explained. The authors guess that irregular laser fiber damage might be based on the homogeneity of laser fiber glass and consistency of laser energy transmission. [16] Ritchie and coworkers performed a benchtop simulation of laser lithotripsy and investigated output differences between unstripped and stripped laser fibers of Lumenis Slimline^TM^ 365 micrometer after 1 minute of lasing. The electron microscopy images showed irregular laser tip degradation that might mean the laser fiber degradation is not homogenous at the tip of laser fibers. [17] One of the probable reasons of this fiber damage can be made by ureteroscopic deflection to the lower pole of the kidney. Forbes and coworkers reported different flexible ureteroscopy damage rates by laser fiber type and Boston Flexiva laser fiber has fewer failures. [18] The authors in the present study used the same fiber and the readers should consider the probable different rates of laser fiber degradation. The irregular laser fiber degradation, so-called ‘double-firing phenomenon’ in the present study can be the common knowledge after further investigations would be performed.

Despite these limitations, the authors believe that this non-industry-sponsored study showed important information that it can reduce unpredictable scope damage and associated repair cost by revealing invisible laser fiber core degradation. The authors tried to get the reliability and reproducibility while the experiments were performed with almost the same techniques in the previous investigations. [19]

## Conclusion

To reduce ‘double-firing’-induced scope damage, it would be sufficient to cut the visible burned fiber portion when using a large caliber fiber. However, the authors recommend that (i) surgeons should cut the 4-7mm tip of the laser fiber after 3 min continuous stone fragmentation, (ii) laser fiber should be cut 1.0 mm longer than the visible jacket burn repeatedly at high-power settings especially with 200 μm fiber, (iii) After cutting the laser fiber, the laser should be checked whether ‘double-firing’ is no more seen. Because there has been no guideline about cutting the tip of the laser fiber, the results with the measurable parameters of the present study are meaningful.

## Supporting information

S1 Data (XLSX)
